# Integrity of the Pericentriolar Material Is Essential for Maintaining Centriole Association during M Phase

**DOI:** 10.1371/journal.pone.0138905

**Published:** 2015-09-25

**Authors:** Mi Young Seo, Wonyul Jang, Kunsoo Rhee

**Affiliations:** Department of Biological Sciences, Seoul National University, Seoul, Korea; National Cancer Institute, NIH, UNITED STATES

## Abstract

A procentriole is assembled next to the mother centriole during S phase and remains associated until M phase. After functioning as a spindle pole during mitosis, the mother centriole and procentriole are separated at the end of mitosis. A close association of the centriole pair is regarded as an intrinsic block to the centriole reduplication. Therefore, deregulation of this process may cause a problem in the centriole number control, resulting in increased genomic instability. Despite its importance for faithful centriole duplication, the mechanism of centriole separation is not fully understood yet. Here, we report that centriole pairs are prematurely separated in cells whose cell cycle is arrested at M phase by STLC. Dispersal of the pericentriolar material (PCM) was accompanied. This phenomenon was independent of the separase activity but needed the PLK1 activity. Nocodazole effectively inhibited centriole scattering in STLC-treated cells, possibly by reducing the microtubule pulling force around centrosomes. Inhibition of PLK1 also reduced the premature separation of centrioles and the PCM dispersal as well. These results revealed the importance of PCM integrity in centriole association. Therefore, we propose that PCM disassembly is one of the driving forces for centriole separation during mitotic exit.

## Introduction

Centrioles duplicate and segregate in a tight link to the cell cycle [1, 2]. During S phase, a procentriole grows next to the mother centriole and no more procentriole is allowed despite the condition permissive to centriole duplication. The procentriole is eventually disengaged and separated from the mother centriole during M phase and both the centrioles are then licensed to go for another round of centriole duplication in the next cell cycle [3, 4]. Therefore, the associated status of centrioles has been considered to be an intrinsic block to the re-duplication of centrioles. Failure in this regulatory mechanism may cause centriole amplification, and thus the formation of multipolar spindle and eventually contributing to the genomic instability [5, 6].

Separase is a caspase-family cysteine protease that is originally recognized for its role in separation of the sister chromatids [7]. It is well known that separase becomes activated in anaphase and cleaves the cohesin complex that holds the sister chromatids together [8]. The same protease is also essential for centriole disengagement and separation. Depletion of separase blocked centriole disengagement in *Xenopus* egg extracts [9]. Cohesin was proposed as a centriole-engagement factor which should be cleaved by separase during mitosis [10, 11]. However, recent works questioned that the cohesin cleavage by separase is not the whole story for induction of centriole disengagement and separation. First, centrioles in separase-null cell lines remained engaged even after mitotic exit but eventually separated during the following S phase [12]. That is, there may be a way that a procentriole can be separated from its mother centriole even if the separase activity is limited. Second, a sperm-derived centriole in the *C*. *elegans* early embryo needed the separase activity for centriole separation in the first mitosis [13]. However, separase was dispensable for centriole separation in the later cleavages [13]. These results reveal that separase is not crucial for centriole separation of all mitotic cells. Finally, an artificial cleavage of SCC1, a component of the cohesin complex, could not induce centriole separation in *Drosophila* embryos [14]. This result suggests that SCC1 degradation may not be a sole event for centriole separation. Rather, separase may have additional substrates for centriole separation in addition to SCC1. In fact, pericentrin, a major component of pericentriolar material (PCM), has been identified as a novel substrate of separase [15, 16]. Therefore, the separase activity is necessary for centriole separation during mitosis, but there exists a detour to undergo centriole separation even in the absence of the separase activity.

It is known that centrioles are prematurely disengaged and uncoupled when cells are arrested at G2 phase for a long period of time [17, 18]. The premature uncoupling of centrioles during G2 arrest is attributed to the temporal activation of the separase by APC/C [18]. Here, we observed that centrioles are also prematurely uncoupled when the cells were arrested at M phase with S-trityl-L-cysteine (STLC). However, the centrioles in STLC-treated cells were prematurely separated even in a separase-depleted condition. A series of our results suggest that it is the PCM which maintains centriole association during a prolonged mitotic arrest.

## Materials and Methods

### Cell culture and drug treatment

HeLa and hTERT-RPE1 cells (American Type Culture Collection, Manassas, VA, USA; 2009) were cultured in DMEM supplemented with 10% FBS and plasmocin (5 μg/ml, Invivogen). U2OS cells (American Type Culture Collection, Manassas, VA, USA; 2007) were cultured in DMEM-F12 supplemented with 10% FBS and plasmocin. The single or double thymidine block and release method was used to synchronize the cell cycle. For double thymidine block, the cells were treated with 2 mM of thymidine for 17–20 h, incubated in fresh medium for 8 h, and were incubated again with thymidine for 17 h. For G2 arrest, the cells were treated with RO3306 (10 μM). The cells were arrested at M phase with STLC (10 μM) and nocodazole (50–200 ng/ml). BI2536 (200 nM) was treated to inhibit PLK1 activity.

### RNAi and transfection

We depleted the endogenous separase, pericentrin or CDC20 proteins with specific siRNAs. The siRNA sequences for separase (*siSeparase*), pericentrin (*siPericentrin*) and CDC20 (*siCDC20*) were 5’-GCU UGU GAU GCC AUC CUG A-3’, 5’-UGG ACG UCA UCC AAU GAG ATT-3’, and 5’-CGA AAU GAC UAU UAC CUG A-3’, respectively. A scrambled *siCTL* (5’-GCA AUC GAA GCU CGG CUA CTT-3’) was used as a control siRNA. The siRNAs were transfected using RNAi max (Invitrogen), according to the manufacturer’s instructions.

### Immunoblot analysis

Cells were lysed with NP40 lysis buffer (50 mM Tris-HCl [pH7.4], 150 mM NaCl, 0.5% NP40, 0.5% Triton-X, 1 mM EDTA, and 1 mM EGTA). The cell lysates (20 μg protein) were loaded onto a SDS-polyacrylamide gel and transferred onto nitrocellulose membranes (GE healthcare). The following antibodies were used as the primary antibodies in the immunoblotting analysis; separase (ab16170, Abcam; 1:500), CDC20 (ab26483, Abcam; 1:1,000), cyclin B1 (sc-245, Santa Cruz; 1:500), CDK1 (#06–141, Upstate; 1:500), PLK1 (ab17057, Abcam; 1:500), and GAPDH (AM4300, Ambion; 1:10,000). Anti-mouse IgG-HRP (A9044, Sigma; 1:10,000) and anti-rabbit IgG-HRP (DL03L, Calbiochem; 1:10,000) were used as the secondary antibodies.

### Immunofluorescence microscopy

The cells were fixed with ice-cold methanol for 10 min., washed with PBS twice, permeabilized with 0.1% Triton X-100 in PBS for 10 min, and then incubated with 3% BSA (w/v) in PBS containing 0.5% Triton X-100. For immunostaining analyses, we used primary antibodies against centrin-2 (#04–1624, Millipore; 1:1,000), CEP135 [19], CP110 [20], γ-tubulin (sc-7396, Santa Cruz; 1:200), pericentrin [21], CEP192 (A302–324A; Bethyl Laboratories, 1:1,000), and CEP215 [22]. The secondary antibodies were anti-rabbit, anti-mouse, and anti-goat IgG antibodies conjugated with AlexaFluor-488 or -594 (A21202, A21203, A21206, A21207, A11058, Life Technologies: 1:1,000). For triple staining, antibodies were fluorescently labeled using a Zenon labeling kit (Zenon Alexa Fluor 647 Rabbit IgG Labeling Kit, Invitrogen). DNA was counterstained with DAPI. Images were captured on Olympus IX51 microscope equipped with a CCD camera (Qiacam fast 1394, Qiamaging), and analyzed with ImagePro 5.0 (Media Cybernetics).

### Chromosome spread

STLC-arrested cells were trypsinized and harvested by centrifugation. Then, 75 mM KCl was added to the cell pellet. After mild resuspension, the cells were incubated further with the hypotonic solution for 20 min at 37°C and centrifuged to remove the buffer. Freshly prepared fixative (Carnoy’s solution; 75% methanol and 25% acetic acid) was added to the pellet and changed three times by centrifuging at 200×g for 5 min. For spreading, the cells were dropped onto glass slides and dried at 50°C, and stained with DAPI.

### 3D-Structured illumination microscopy (SIM)

3D-SIM images were acquired using ElyraPS1 microscope system (Carl Zeiss) which is located at the National Center for Inter-University Research Facilities (Seoul National University). The SIM system achieves a resolution of 100 nm along the x-y axis and 300 nm along the z axis. Laser lines at 405, 488 and 561 nm were used for excitation. For channel alignment, we used multispec calibration slide (170 nm beads, cat. No. 1783–455, Carl Zeiss). The SIM images (15 images with five different phases for 3 different rotation of illumination grid for each SIM image) were acquired with an EMCCD camera (Andor Technology) (512x512 pixels) and processed with Zen (Carl Zeiss) software.

## Results

### Paired centrioles are prematurely separated in STLC-treated cells

**Fig 1:**
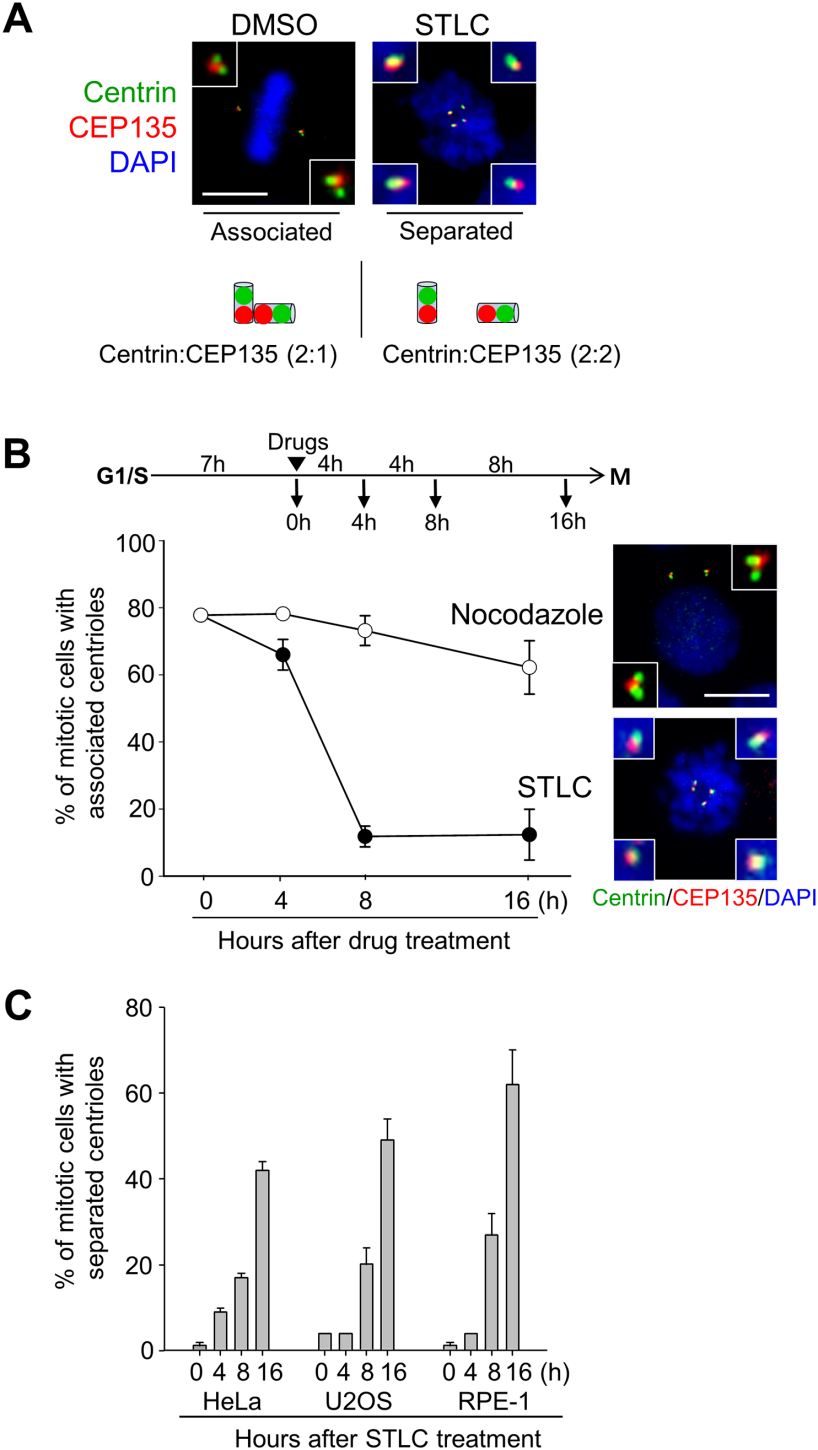
Premature loss of centriole association in STLC-arrested cells. (A) After double thymidine block and release, HeLa cells were treated with DMSO or STLC (10 μM) and immunostained with the centrin-2 (green) and CEP135 (red) antibodies. DNA was stained with DAPI (blue). Scale bar, 10 μm. As illustrated, centriole configuration was determined by the ratio of centrin-2 and CEP135 foci. (B) HeLa cells were synchronously released from G1/S phase, treated with STLC or nocodazole, and cultured for the indicated time periods. Centriole association was determined with the centrin-2 and CEP135 antibodies. The experiments were repeated 3 times with over 150 cells at each time point. (C) Asynchronous HeLa, U2OS and RPE-1 cells were treated with STLC (5 μM) for the indicated time periods and immunostained with the centrin-2 and CEP135 antibodies. The mitotic cells with separated centrioles were counted. The experiments were repeated twice with over 100 cells at each time point. Values are means and standard deviations.

During S phase, a procentriole is orthogonally assembled to the mother centriole, which may be determined with electron microscope [12, 23] Once cells enter M phase, procentrioles are disengaged from mother centrioles, but may still remain closely associated near the mother centrioles. The procentrioles are eventually separated from the mother centrioles at the end of mitosis. Centriole association and separation may be distinguished with a fluorescent microscope, based on the distance between the centriole markers, such as centrin-2. Markers for proximal centrioles, such as C-NAP1 and CEP135, may be used to have a better idea for centriole association. The proximal markers were frequently overlapped in associated centrioles (Fig 1A).

We investigated centriole configuration in the M phase-arrested cells. We initially used STLC, an inhibitor of EG5, to examine centriole behavior in M phase arrested cells. EG5 is a mitotic kinesin which coordinates the spindle pole separation in early mitosis. Therefore, the STLC treatment blocked bipolar spindle formation, without affecting the microtubule stability [24]. The time course experiments revealed that the centrioles in the STLC-treated cells were mostly separated in 8 hours (Fig 1B). We also treated HeLa cells with nocodazole, a microtubule destabilizer [25]. The results showed that most of the centrioles in the nocodazole-treated cells remained associated in 8 hours (Fig 1B). The number of cells with separated centrioles slightly increased at 16^th^ hour after the nocodazole treatment, but some of the cells did not look healthy with signs of mitotic slippage and apoptosis (Fig 1B). Premature centriole separation was also observed upon the STLC treatment in other cell lines, such as RPE-1 and U2OS (Fig 1C). These results reveal that centrioles are prematurely separated in cells whose cell cycle is arrested at M phase by STLC for a prolonged time period.

### Failure of centriole association in STLC-treated cells was independent of the separase activity

**Fig 2:**
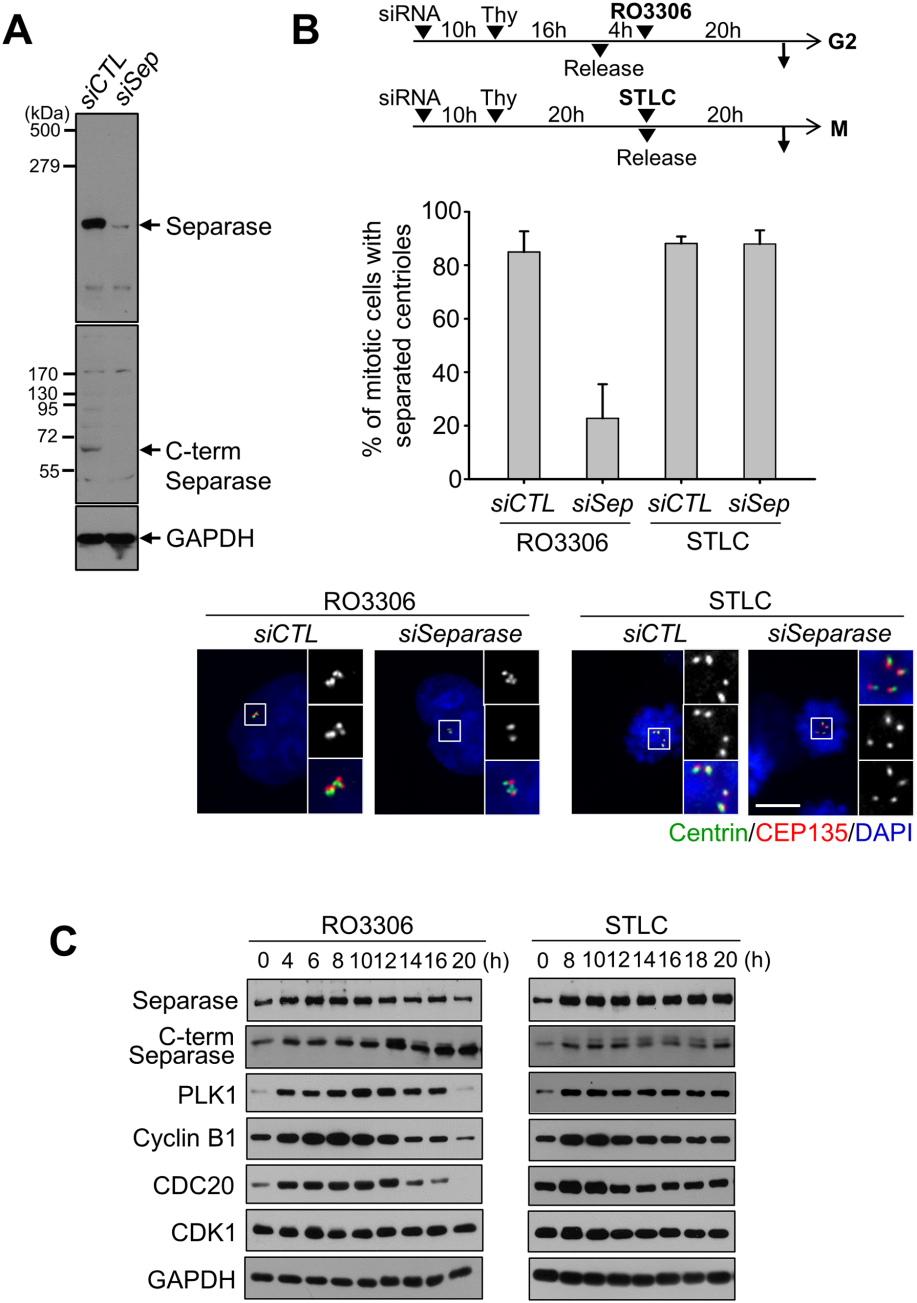
Premature centriole separation in separase-depleted cells. (A) Immunoblot analysis was performed to determine cellular levels of the full-length and C-terminal fragment of separase in the *siSeparase*-transfected HeLa cells. (B) The *siCTL*- or *siSeparase*-transfected HeLa cells were enriched to S phase with a single thymidine block and release, and treated with RO3306 or STLC for 20 h. The cells were fixed and coimmunostained with the centrin-2 (green) and CEP135 (red) antibodies. Based on the ratio of centrin-2 and CEP135 foci, the number of cells with separated centrioles was counted. Scale bar, 10 μm. The experiments were repeated 3 times with over 100 cells at each group. Values are means and standard deviations. (C) HeLa cells were synchronously released from G1/S phase in the presence of RO3306 (10 μM) or STLC (10 μM). The cell lysates at the indicated time points were subjected to immunoblot analyses with the indicated antibodies.

Separase is known to play a critical role in centriole disengagement and separation during mitotic exit [3, 12]. Premature centriole separation has been previously reported in the G2 phase-arrested cells with RO3306, a CDK1 inhibitor [17, 18] and the event was separase-dependent. In order to test whether separase is also essential for premature centriole separation in M phase-arrested cells, we depleted endogenous separase with siRNA transfections and treated the cells with STLC (Fig 2A and 2B). The RO3306-treated cells were included as a control to determine a residual activity in the separase-depleted cells. The results showed that depletion of separase prevented the G2-arrested cells from premature centriole separation (Fig 2B). On the other hand, centrioles in the M phase-arrested cells were still separated even in the same separase–depleted condition (Fig 2B).

We compared premature centriole separation in between the RO3306- and STLC-treated cells. A time-course analysis revealed a surge of the active form of separase at the 12^th^ hour followed by reduction of the cyclin B1 and CDC20 levels in RO3306-treated cells (Fig 2C). These results are consistent with the previous notion that the APC/C activity surges when the cells are arrested at G2 phase for a prolonged time period [18]. In contrast, the cellular separase along with cyclin B1 and CDC20 levels remained constant in the STLC-treated cells (Fig 2C). These results suggest no APC/C surge in the M phase-arrested cells. Premature centriole separation in the STLC-treated cells might occur in a mechanism different from that in the RO3306-treated cells.

**Fig 3:**
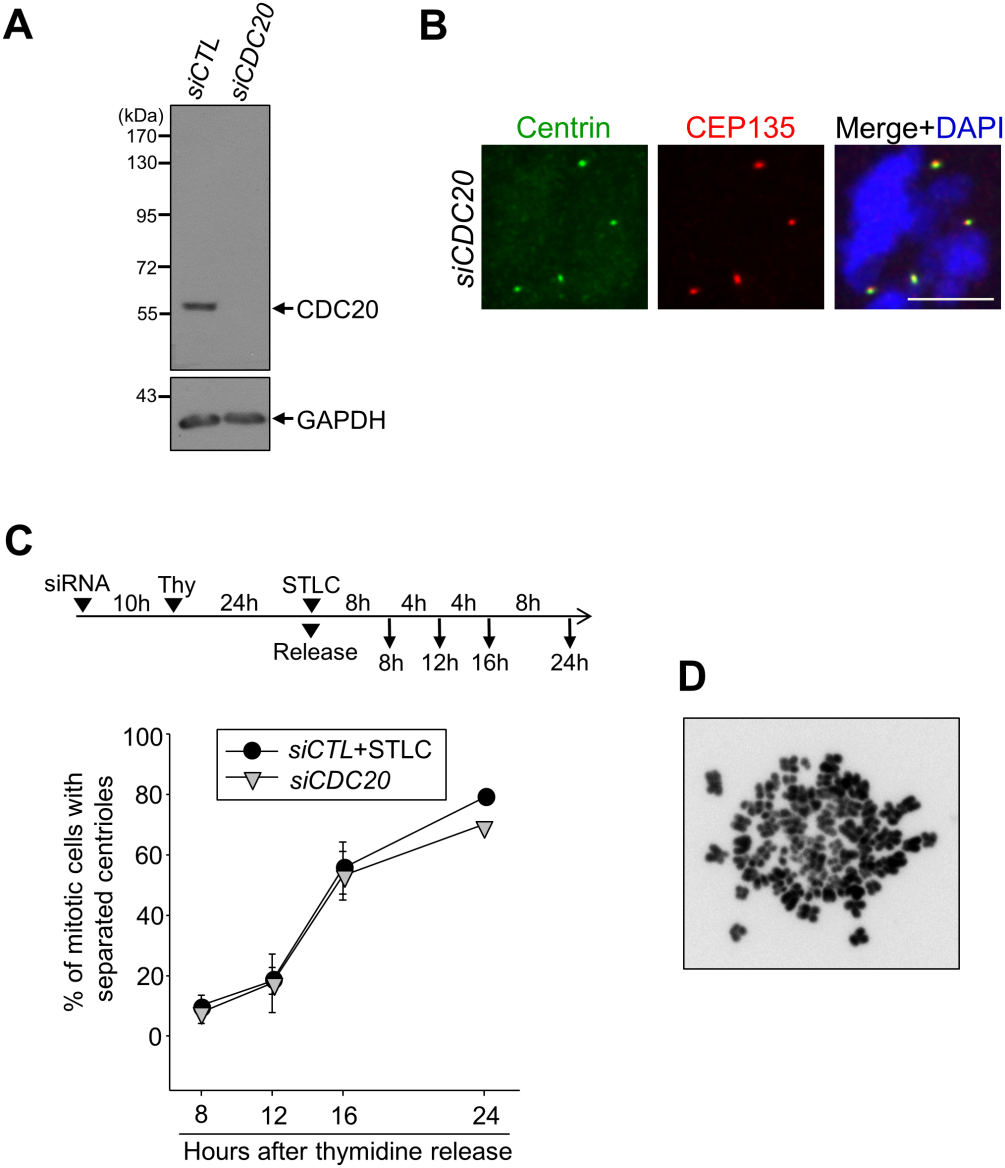
Premature centriole separation in CDC20-depleted cells. (A) Immunoblot analysis was performed to determine the CDC20 levels in *siCDC20*-transfected RPE-1 cells. (B) The CDC20-depleted mitotic cells were immunostained with antibodies specific to centrin-2 (green) and CEP135 (red). Scale bar, 10 μm. (C) The CDC20-depleted RPE-1 cells were synchronized with a thymidine block and release, and fixed for immunostaining at the indicated time points. The control cells were treated with STLC for M phase arrest. The number of mitotic cells with separated centrioles was counted at each time points. Experiments were repeated three times with over 150 cells per each group. Values are means and standard deviations. (D) HeLa cells were treated with the STLC (10 μM) for 20 h and their chromosomes were spread and stained with DAPI.

CDC20 is a co-activator for APC/C. Its depletion blocks the cell cycle prior to metaphase with little APC/C activity, thus separase remains inactive [26]. We observed that most of the CDC20-depleted RPE-1 cells possessed separated centrioles (Fig 3A and 3B). We performed a time-course experiment to compare the frequencies of centriole separation between the CDC20-depleted cells and STLC-treated cells. The results showed that centrioles were separated in both the CDC20-depleted cells and STLC-treated cells to the similar extent over time (Fig 3C). Over a half of centrioles were separated at 16th hour after the G1/S release. These results provide additional evidence that failure of centriole association in M phase-arrested cells is independent of the separase activity.

A prolonged metaphase arrest is known to induce asynchronous separation of the sister chromatids [27, 28]. This phenomenon, called ‘cohesion fatigue’, may occur even in the separase-depleted cells [27, 28]. In order to examine whether centrioles are prematurely separated in association to cohesion fatigue, we performed chromosome spread experiments in the STLC-treated cells. As shown in Fig 3D, the sister chromatids were tightly attached to each other, indicating that loss of centriole association in STLC-treated cells is not linked to the cohesion fatigue phenomenon.

**Fig 4:**
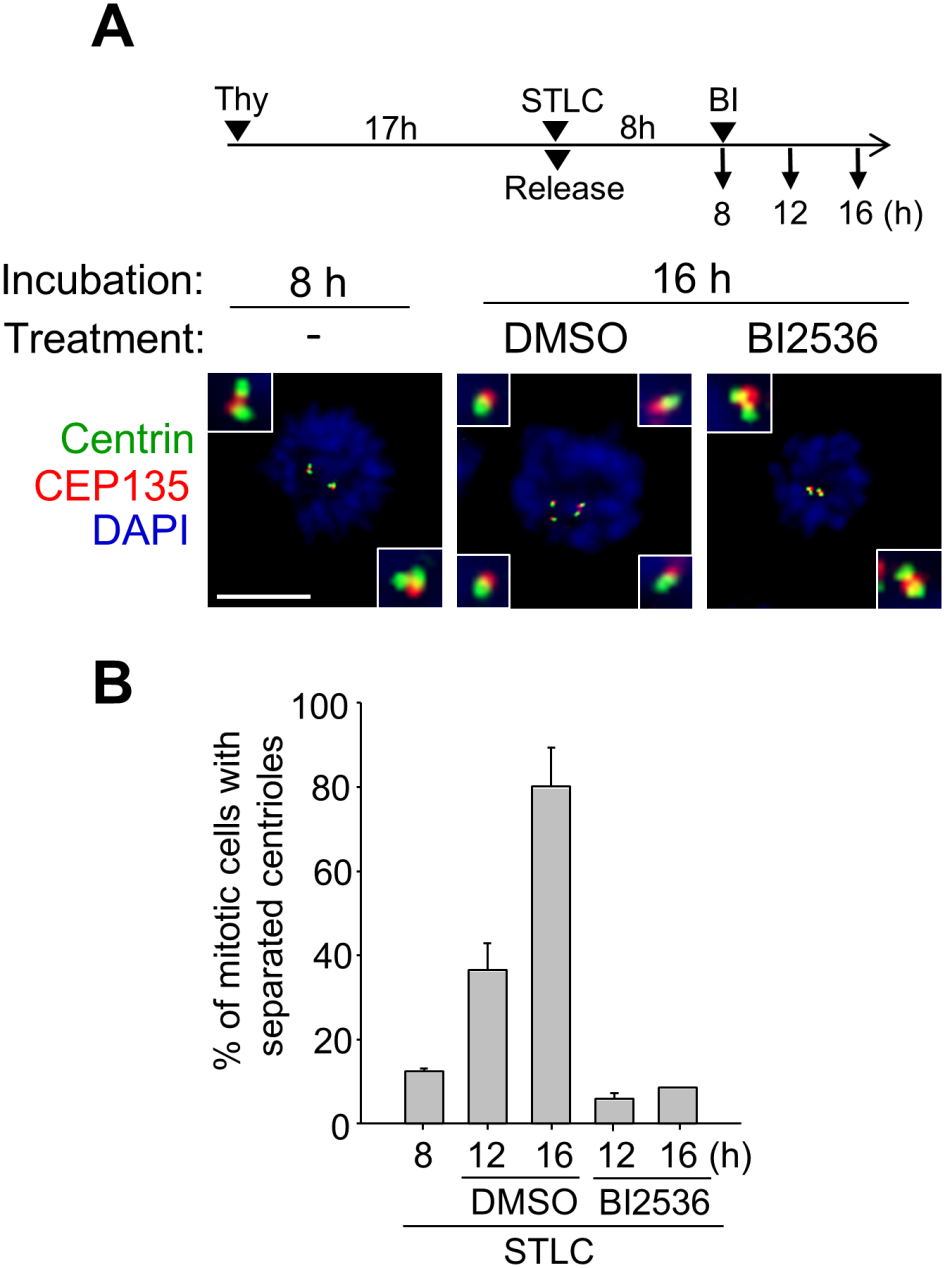
Effects of BI2536 on the premature centriole separation. (A) The RPE-1 cells were treated with STLC (10 μM) as soon as they were released from a single thymidine block. Eight hours later, the cells were treated with BI2536 (200 nM) for the indicated time periods, and immunostained with the centrin-2 (green) and CEP135 antibodies (red). Scale bar, 10 μm. (B) The number of cells with separated centrioles were counted. The experiments were repeated three times, with over 150 cells at each group. Values are means and standard deviations.

The PLK1 activity is critical for centriole disengagement and separation during mitosis [12, 29]. In fact, centrioles remained engaged when both the separase and PLK1 activities were blocked [12]. We used BI2536, a PLK1 inhibitor, to examine the involvement of the PLK1 activity in the premature centriole separation of the M phase-arrested cells (Fig 4A and 4B). The results showed that the number of cells with separated centrioles was significantly reduced when the cells were treated with BI2536 along with STLC (Fig 4A and 4B). These results implicate that the PLK1 activity is required for the association of centriole pair in the M phase-arrested cells.

### A high dose of nocodazole inhibits the premature separation of centrioles

**Fig 5:**
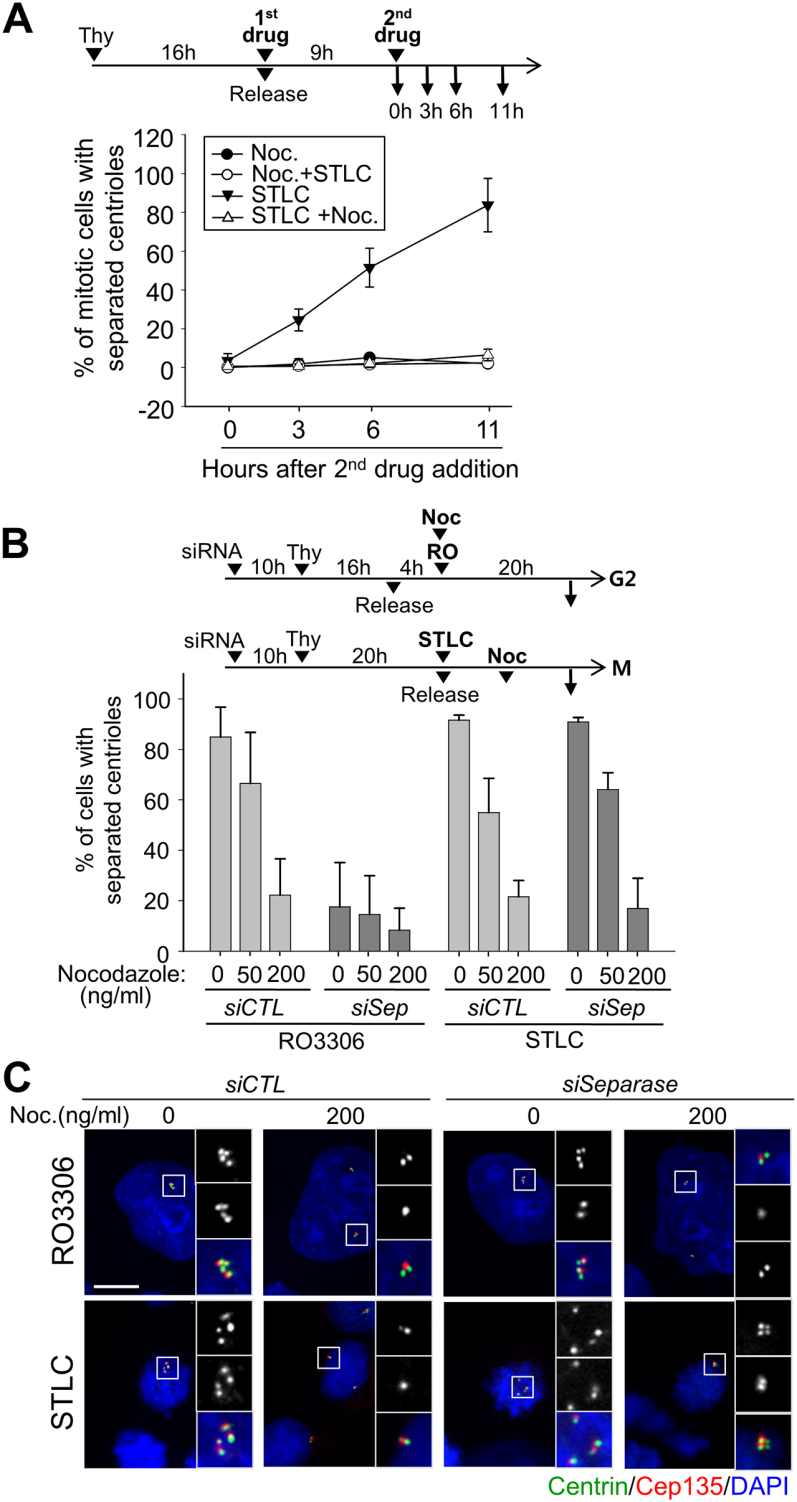
Effects of nocodazole on the premature centriole separation. (A) HeLa cells were synchronously released from G1/S phase in the presence of nocodazole or STLC. Nine hours later, the second drug was added and cultured for the indicated time periods. The cells were immunostained with the centrin-2 and CEP135 antibodies and the number of cells with separated centrioles was counted. The experiments were repeated twice with over 100 cells at each group. Values are means and standard deviations. (B) The separase-depleted HeLa cells were treated with RO3306 or STLC for 20 h. Nocodazole (0, 50 or 200 ng/ml) was added along with RO3306 or at the 9^th^ hour after the STLC treatment. The cells with separated centrioles were counted and statistically analyzed as above.

We treated the HeLa cells in combinations of STLC and nocodazole to compare their reciprocal effects with regard to the centrosome. As expected, separated centrioles were observed in over 80% of the STLC-treated cells (Fig 5A). However, centrioles were not scattered with the nocodazole treatment even for 20 h (Fig 5A). Furthermore, addition of nocodazole at 9^th^ hour after the STLC treatment prevented paired centrioles from being dispersed (Fig 5A). On the other hand, addition of STLC to the nocodazole-treated cells did not induce premature centriole separation. These results suggest that nocodazole has an inhibitory effect on the premature separation of centrioles in M phase-arrested cells.

To further investigate the inhibitory effects of nocodazole on premature loss of centriole association, we treated the G2 or M phase-arrested cells with a high and low dose of nocodazole (200 and 50 ng/ml, respectively). The results showed that nocodazole significantly reduced the incidence of centriole separation in a concentration-dependent manner in both RO3306- and STLC-treated cells, irrespective of the separase activity (Fig 5B). These observations suggest that the microtubule depolymerizing activity of nocodazole might let the centriole pair remain associated.

**Fig 6:**
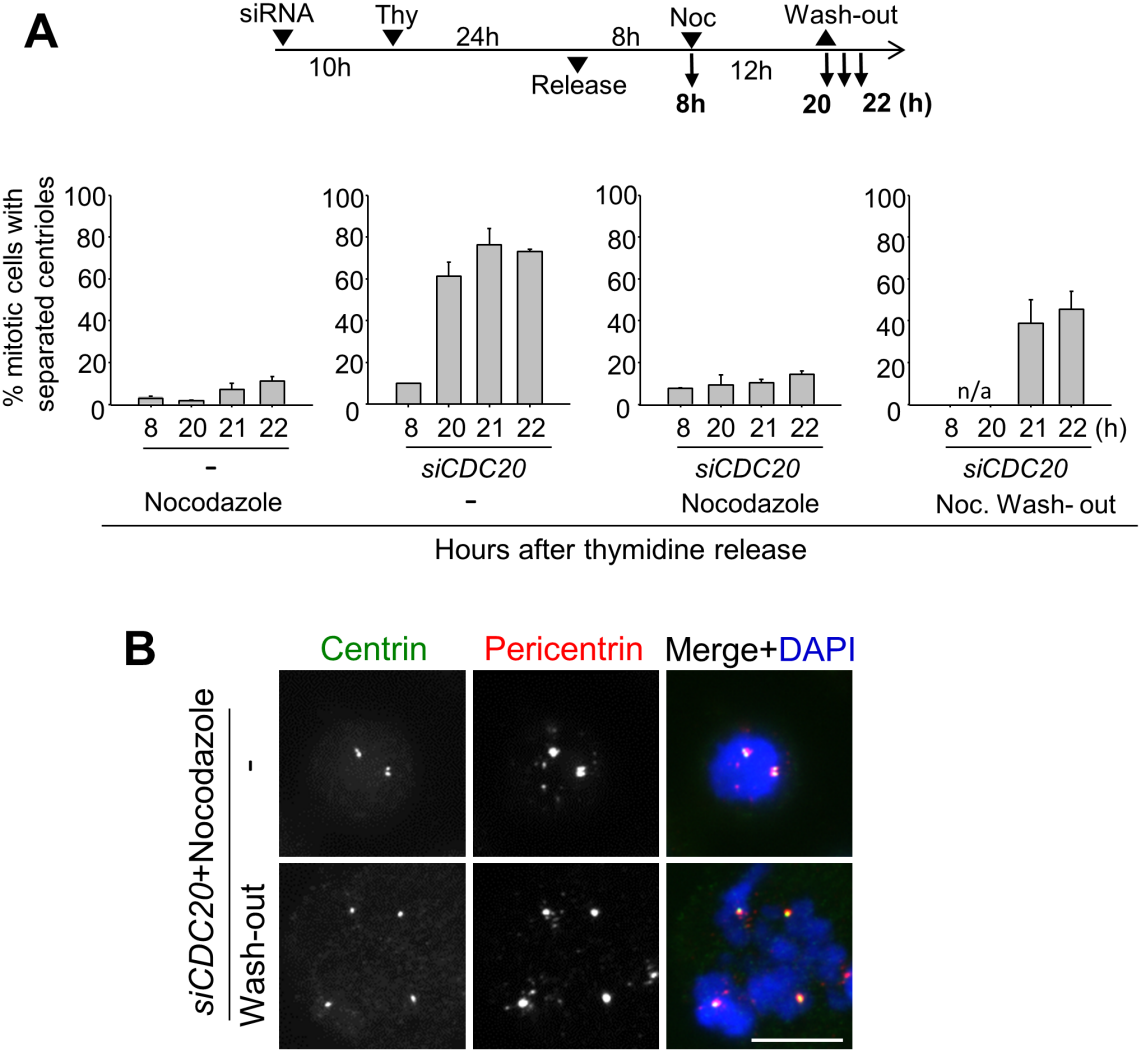
Induction of the premature centriole separation after the removal of nocodazole. (A) The CDC20-depleted RPE-1 cells were synchronously released from a single thymidine block. Nocodazole (200 ng/ml) was treated for 12 h and then washed out. At indicated time points, the cells were fixed for immunostaining with the centrin-2 and CEP135 antibodies to determine centriole separation. The experiments were repeated 3 times with over 100 cells at each group. Values are means and standard deviations. (B) Representative immunostaining results of the CDC20-depleted RPE-1 cells with or without nocodazole wash-out. The cells were immunostained with the centrin-2 (green) and pericentrin (red) antibodies. Scale bar, 10 μm.

We further examined nocodazole effects in CDC20-depleted cells. As shown before, a high dose of nocodazole arrested the cell cycle at prometaphase without centriole scattering (Figs 1B and 5A). CDC20 depletion also arrested the cell cycle at M phase and most centrioles were scattered at 20th hour after the S phase release (Figs 3C and 6A). However, in the presence of a high dose of nocodazole, centrioles remained associated even in the CDC20-depleted cells (Fig 6A). Furthermore, centrioles quickly started to separate along with a PCM protein, pericentrin, as soon as nocodazole was removed from the medium (Fig 6B). Based on the results, we propose that the microtubules might be pulling centrioles apart to make them lose their association in M phase-arrested cells.

### PCM is critical for the maintenance of centriole association

**Fig 7:**
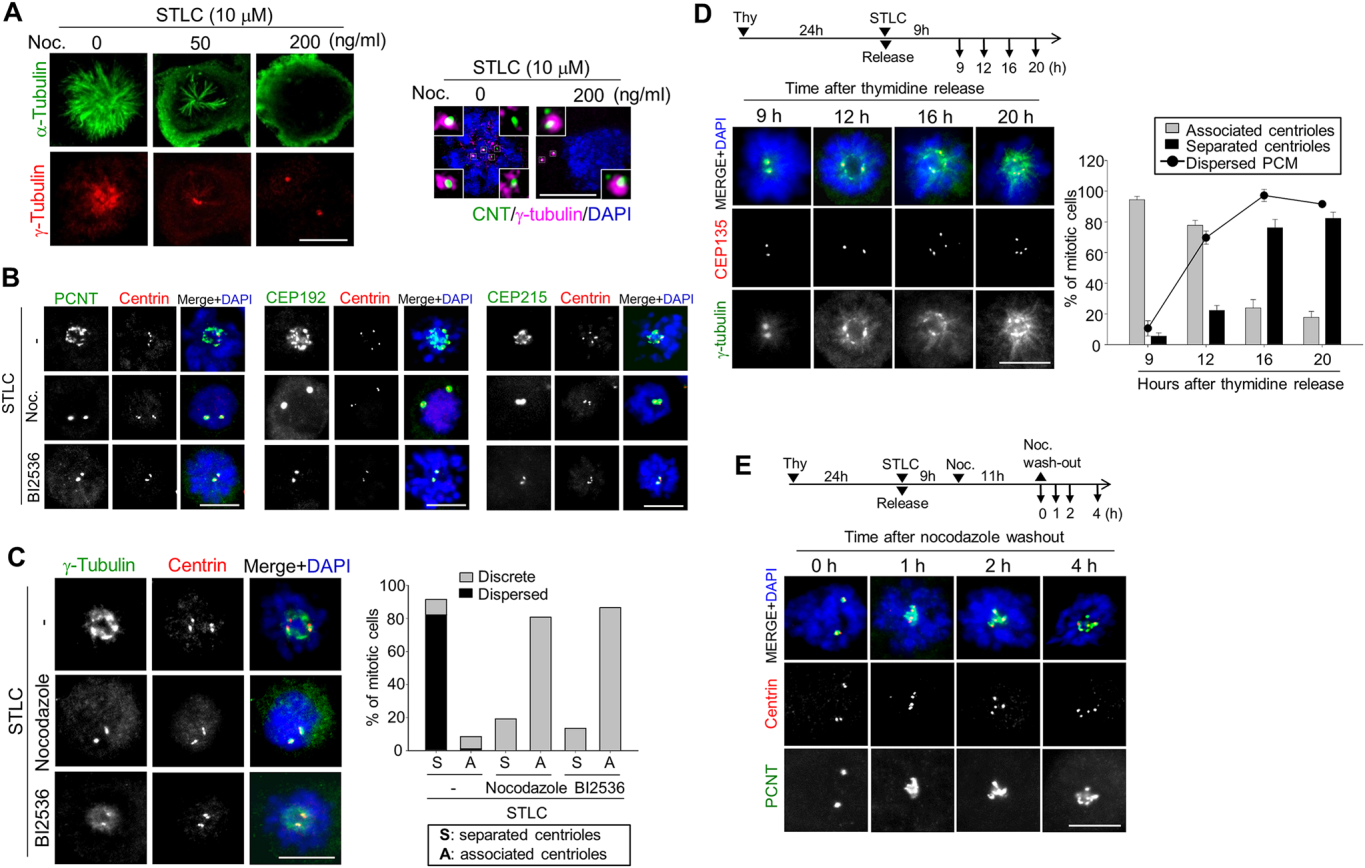
Effects of nocodazole and BI2536 on PCM dispersal in STLC-treated cells. (A) HeLa cells were cultured in the presence of STLC for 20 h. Nocodazole was added at the 9^th^ hour point. The cells were immunostained with the α-tubulin (green) and γ-tubulin (red) antibodies. The cells were also immunostained with centrin-2 and CEP135 antibodies to determine centriole association. Scale bar, 10 μm. (B, C) HeLa cells were treated with STLC for 20 h. Nocodazole (200 ng/ml) or BI2536 (200 nM) was added at 9^th^ h. The cells were immunostained with antibodies specific to pericentrin, CEP192, CEP215 and γ-tubulin (green) along with centrin-2 (red). Scale bar, 10 μm. (C) The γ-tubulin staining patterns were categorized as discrete and dispersed. The experiments were repeated twice, with over 150 cells at each group. Values are means and standard deviations. (D) HeLa cells were arrested at S phase with thymidine and released in the presence of STLC. At the indicated time points, the cells were coimmunostained with antibodies specific to CEP135 and γ-tubulin to determine centriole association and PCM dispersal, respectively. The experiments were repeated twice, with over 100 cells at each group. Values are means and standard deviations. (E) HeLa cells were treated with STLC for 9 h and then with nocodazole for 11 h. The cells were then transferred to a nocodazole-free medium and cultured for up to 4 h. At the indicated time points, the cells were coimmunostainined with antibodies specific to centrin-2 and pericentrin. DNA was stained with DAPI.

Effects of nocodazole on the cellular microtubule network were visualized with the α-tubulin and γ-tubulin antibodies. STLC made mitotic spindles a radial pattern since it inhibited bipolar spindle formation (Fig 7A) [24]. At the same time, the γ-tubulin signals became dispersed in STLC-treated cells (Fig 7A) Addition of nocodazole reduced the radial microtubules in a dose-dependent manner, so that a high dose of nocodazole almost completely abolished microtubules in the cell (Fig 7A). The γ-tubulin signals looked discrete and intense at spindle poles in the nocodazole-treated cells (Fig 7A). These results suggest that nocodazole effectively reduced the microtubule pulling force around centrosomes and eventually inhibited premature loss of centriole association in STLC-treated cells.

We, then, examined PCM proteins in M phase-arrested cells to look for a correlation between the PCM structure and centriole association. The HeLa cells were treated with STLC for M phase-arrest and immunostained with antibodies for PCM proteins, such as pericentrin, CEP192 and CEP215. The results showed that all tested PCM proteins were dispersed along with scattered centrioles in STLC-treated cells (Fig 7B). Addition of nocodazole and BI2536 made PCM proteins discrete around the paired centrioles (Fig 7B). In order to examine relationship between PCM dispersal and centriole scattering, we coimmunostained the cells with the γ-tubulin and centrin-2 antibodies. The results showed that γ-tubulin was dispersed in the most of the STLC-treated cells with scattered centrioles (Fig 7C). The γ-tubulin pattern became discrete around the paired centrioles when nocodazole and BI2536 was added in the medium (Fig 7C). These results suggest that dispersal of PCM is linked to the maintenance of centriole association in M phase-arrested cells.

In order to have a clue whether the dispersed centrosomal proteins in STLC-treated cells originated from the centrosomal PCM or not, we performed time-course experiments (Fig 7D and 7E). HeLa cells were synchronized with a single thymidine block and released in the presence of STLC. The cells were fixed and coimmunostained with the CEP135 and γ-tubulin antibodies at the indicated time points. The number of CEP135 foci indicates the status of centriole association. At 9th hour after the STLC treatment, most of the prometaphase cells showed discrete two γ-tubulin foci along with associated centrioles (Fig 7D). PCM dispersal initiated at 12th hour, but most of centrioles remained associated (Fig 7D). However, most of centrioles were separated as the mitotic block extended (Fig 7D). These results suggest that the PCM dispersal precedes the premature centriole separation in STLC-treated cells.

The HeLa cells were co-treated with STLC and nocodazole for 20 h and transferred to a nocodazole-free medium. The cells were further cultured for up to 4 h, harvested at the indicated time points, and coimmunostained with antibodies specific to centrin-2 and pericentrin. The results showed that the immunostained area of pericentrin was initially limited to the centrosome but expanded from it (Fig 7E). Taken together, these results indicate that the dispersed PCM in the STLC-treated cells may originate from the centrosome.

**Fig 8:**
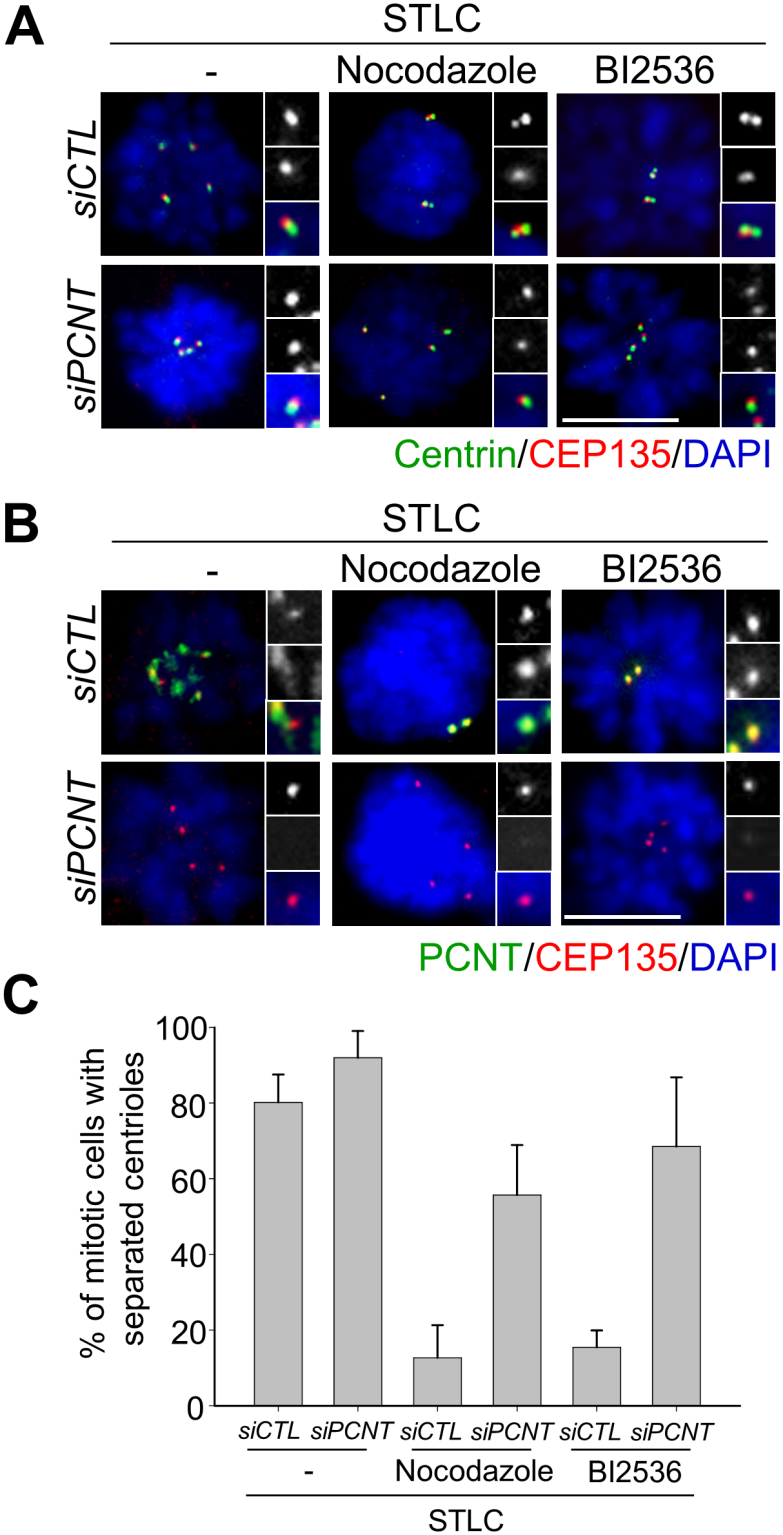
Depletion of pericentrin enhanced the premature separation of centrioles at M phase-arrested cells. (A) Pericentrin-depleted HeLa cells were synchronized with a single thymidine block and release, and treated with STLC. Nine hours later, nocodazole or BI2536 was added and cultured for 11 h. The cells were coimmunostained (A) with centrin-2 (green) and CEP135 (red) antibodies to determine centriole association, (B) and with pericentrin (green) and CEP135 (red) antibodies to determine PCM. Scale bar, 10 μm. (C) The experiments were repeated three times with over 100 cells per group and statistically analyzed. Values are means and standard deviations.

In order to determine the importance of PCM integrity around the centrioles in the centriole association, we depleted pericentrin, a PCM scaffold protein, and looked into the centrioles in nocodazole- or BI2536-treated cells. Surprisingly, the number of cells with separated centrioles significantly increased even in the presence of nocodazole and BI2536 (Fig 8). These results support the notion that the PCM integrity is important for maintaining centriole association in M phase-arrested cells.

### Centriole configurations in the nocodazole- and BI2536-treated cells by 3D-SIM

**Fig 9:**
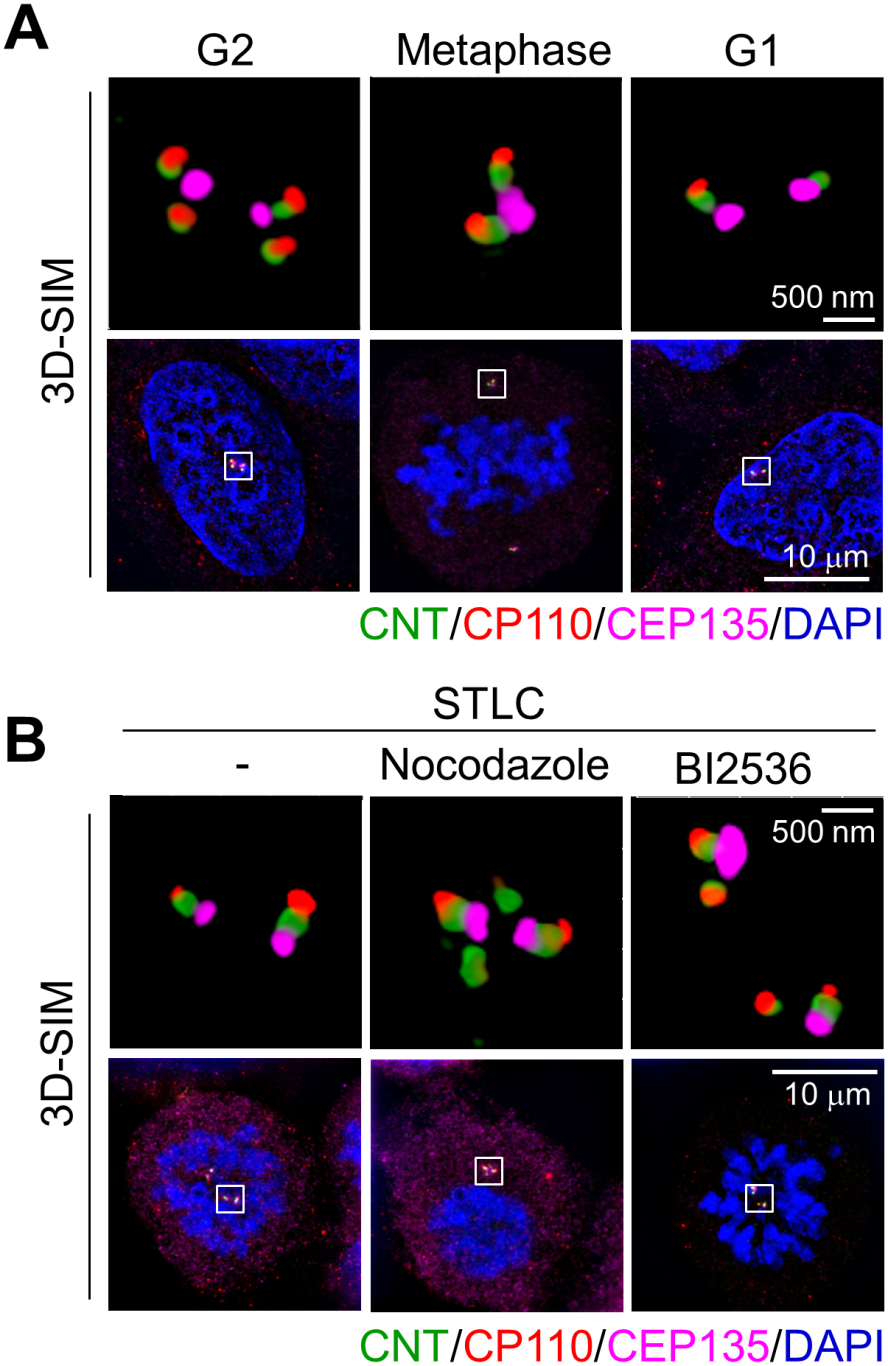
Centriole configurations in STLC-, nocodazole- and BI2536-treated cells by 3D structured illumination microscopy. (A) G2, metaphase and G1 phase cells were obtained from synchronized populations of HeLa cells. The cells were fixed and coimmunostained with antibodies specific to centrin-2 (green), CEP135 (pink) and CP110 (red) to determine centriole configurations. (B) HeLa cells were synchronously released from a single thymidine block in the presence of STLC. Nine hours after the release, nocodazole or BI2536 was added. Eleven hours later, the M phase arrested cells were coimmunostained with the antibodies specific to centrin-2, CEP135 and CP110. Scale bars, 500 nm and 10 μm.

Centrioles in the nocodazole-treated cells revealed the 2:1 configuration of centrin-2 and CEP135 and, therefore, were determined as ‘associated’. We used super-resolution microscrope to further investigate centriole configurations in M phase-arrested cells. The centrioles were immunostained with triple antibodies specific to CEP135 (a proximal centriole marker), centrin-2 (a centriole marker), and CP110 (a distal centriole marker). The results showed that the centrioles in G2 phase cells showed the 2:1 ratio of centrin-2 and CEP135 (Fig 9A). The 2:1 ratio was also observed in the centrioles of metaphase cells, but the CEP135 signal was an overlap of two distinct dots (Fig 9A). Furthermore, the centrioles in nocodazole- and BI2536-treated cells looked similar to those in metaphase cells (Fig 9B). However, it was hard to determine whether two centrioles were orthogonally associated or not. The centrioles in G1 phase cells and STLC-treated cells clearly looked 2:2 ratio of centrin and CEP135 (Fig 9A and 9B). The 3D-SIM analysis confirmed that the mother and daughter centrioles were closely associated in nocodazole- and BI2536-treated cells, but they are separated in STLC-treated cells.

## Discussion

In early days, Mazia, Borisy and others observed that a prolonged mitotic arrest induced dispersal of spindle poles in sea urchin embryos and CHO cells [30–32]. The dispersal of spindle poles can result in multipolar spindle formation with each pole containing a single centriole (reviewed in [33]). In the present work, we also observed premature loss of centriole association in M phase-arrested cells. However, this phenomenon was selective to the cell cycle blocking agents: It was efficiently induced by STLC, but not with nocodazole within a limited time period. This led us to dissect sequential events of centriole association and separation during mitosis.

A prolonged arrest of cell cycle at G2 phase results in premature separation of centrioles [17, 18]. It is also known that the S phase-arrest of the CHO and U2OS cells results in centriole separation, followed by centriole over-duplication [34–36]. Therefore, it is likely that procentrioles are separated from the mother centrioles once the cell cycle is arrested at any interphase stage for a prolonged time period [17, 18, 35, 36]. However, an associated status of centrioles may not be the same between the G2 phase- and M phase-arrested cells.

**Fig 10:**
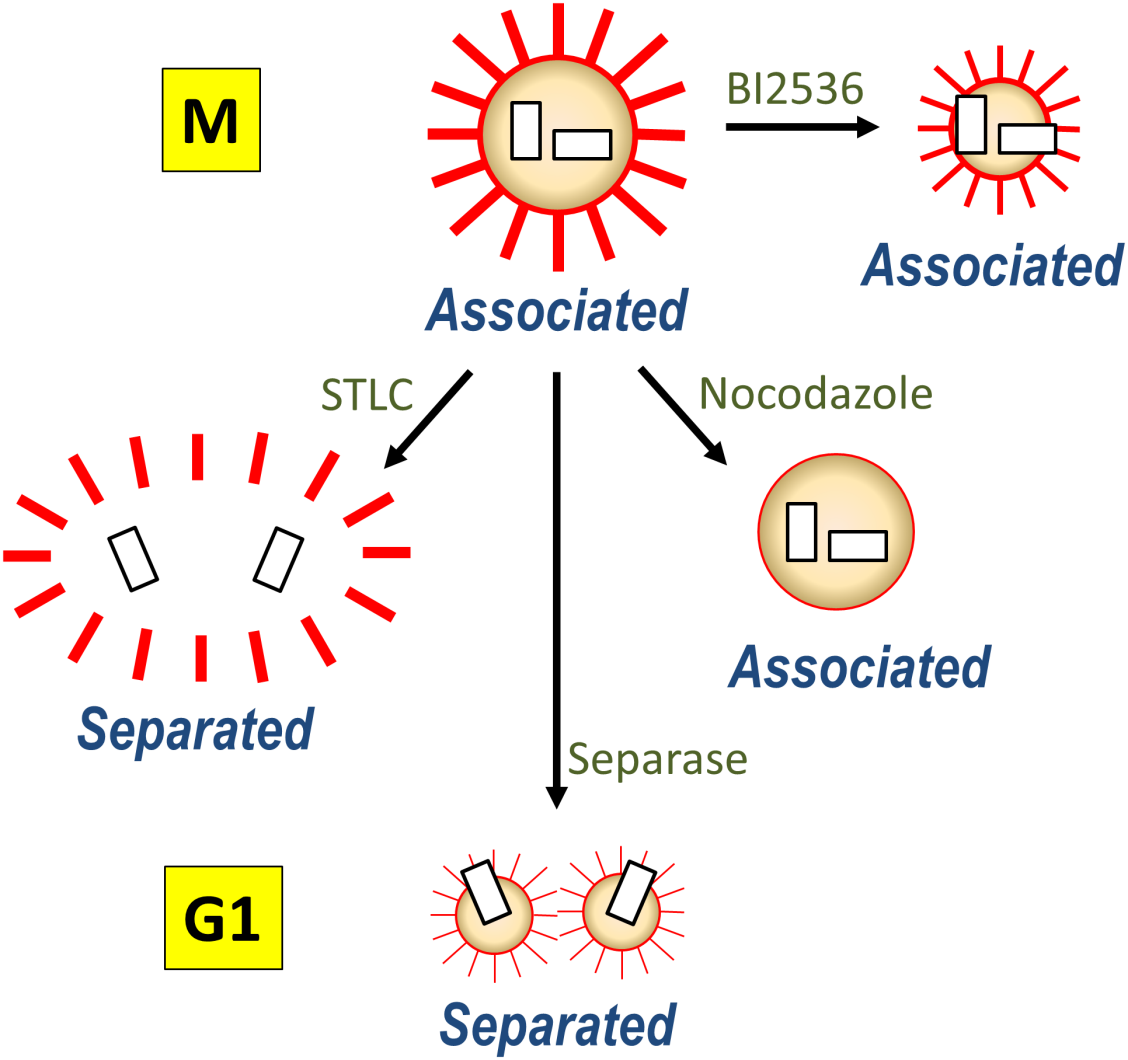
A model of centriole separation in M phase-arrested cells. Associated centrioles in M phase are separated as soon as the cell exit mitosis. Separase plays an essential role in centriole separation. The STLC treatment arrests the cells at M phase, and induces the premature centriole separation along with PCM dispersal. The nocodazole treatment also arrests the cells at M phase, but the centrioles remain associated and PCM looks intact without microtubule emanation. The BI2536 treatment keeps the integrity of PCM around the centriole pair, suggesting that PLK1 is also involved in the PCM dispersal in M phase-arrested cells.

The separase activity is reported to be essential for the premature centriole separation in G2 phase-arrested cells [18]. Untimely activation of APC/C in the G2 phase-arrested cells leads to securin degradation and thus release of the active separase, which is held accountable for the premature separation of centrioles [18]. It remains to be identified what are the substrates of separase for premature centriole separation in G2 phase-arrested cells. In case of M phase-arrested cells, however, the centrioles underwent separation even if separase was depleted. This observation was enforced with the CDC20-depleted cells. We propose the following possibilities to resolve how the centrioles in M phase-arrested cells are prematurely separated in a separase-independent manner. First, substrates of separase responsible for centriole association were removed from the centrosome during the prolonged M phase arrest. PLK1 might take a part in removal of the substrates from the centrosome during the prolonged M phase arrest in a similar manner to dissociate cohesin from the prophase chromosomes [10, 11]. As a result, the centriole pairs remained associated to each other in BI2536-treated cells (Fig 10). Second, procentrioles were torn off from mother centrioles with another force which can override depletion of separase. We propose that microtubule pulling force might be such a force for induction of centriole separation and scattering in M phase-arrested cells (Fig 10). Both the mechanisms are not exclusive to each other but may happen simultaneously (Fig 10).

Importance of microtubule pulling force in centriole separation has been suggested in the early divisions of *C*. *elegans* embryos [13]. Here, we observed that the frequency of centriole scattering in M phase-arrested cells was significantly reduced with nocodazole of which the concentration totally disrupted mitotic spindles. Dispersal of PCM around separated centrioles is another piece of evidence that the PCM structure is critical for the maintenance of centriole association in M phase. We interpret that nocodazole removed the microtubule pulling force around the centrioles and, as a result, prevented PCM from dispersal in M phase-arrested cells. Consistent with our view, the PCM dispersal was readily visible from the centrosome as soon as nocodazole was washed out from the STLC-containing medium (Fig 7E). The microtubule pulling force might be also essential for centriole separation in G2 phase-arrested cells, since centriole separation was inhibited with the nocodazole treatment (Fig 5B).

We observed that a high dose of nocodazole (200 ng/ml) had a preventative effect on centriole separation, whereas a low dose of nocodazole (50 ng/ml) caused centriole scattering over a prolonged mitotic block. This result is consistent with a previous report that the colcemid treatment induced a mitotic delay with the centrioles disoriented in 2:2:0 or 2:1:1 pattern [31]. We interpret that any prolonged mitotic arrest might cause the premature loss of centriole association as far as a residual amount of microtubules are present near the spindle poles. In support of this view, it was reported that the cells with monopolar spindles were predominant with a high dose of nocodazole [32].

PLK1 is known to be required for centriole disengagement and separation [12, 29]. Despite its importance, the role of PLK1 in regulating centriole disengagement and separation has been ambiguous. Our results revealed that BI2536 made PCM remain discrete even in the M phase-arrested cells (Fig 7B and 7C). This allowed us to propose that PLK1 is also essential for PCM disintegration during mitotic exit (Fig 8). In fact, it was suggested that the imbalance of phosphorylation and de-phosphorylation of PCM proteins might be the underlying mechanism for the PCM disassembly at the end of mitosis, along with the cortical forces [37]. One of the candidate PCM proteins is pericentrin which is cleaved by separase during mitotic exit [15, 16].

Our works provide evidence that the PCM integrity is linked to maintain an associated state of the centrioles. Microtubule pulling force and PLK1 may induce PCM disassembly in M phase-arrested cells, independent of the separase-mediated changes in the centrosome (Fig 10). Therefore, we propose that this PCM disassembly is one of the driving forces for centriole separation during mitotic exit.
